# Interpretable machine learning models to predict venous thromboembolism in sepsis patients with type 2 diabetes mellitus

**DOI:** 10.3389/fendo.2026.1958305

**Published:** 2026-09-30

**Authors:** Longyang Ma, Xiaoyuan Yu, Qi Xin

**Affiliations:** 1 Department of Emergency Surgery, Shaanxi Provincial People’s Hospital, Xi’an, China; 2 Department of Hematology, The Affiliated Hospital of Northwest University, Xi’an No. 3 Hospital, Xi’an, China

**Keywords:** machine learning, sepsis, SHAP, type 2 diabetes mellitus, venous thromboembolism

## Abstract

**Background:**

Sepsis patients with type 2 diabetes mellitus (T2DM) face a substantially elevated risk of venous thromboembolism (VTE), yet prediction tools tailored for this population are lacking. This study aimed to develop and validate interpretable machine learning models for predicting in-hospital VTE in septic patients with T2DM.

**Methods:**

A total of 648 sepsis patients with T2DM admitted to Shaanxi Provincial People’s Hospital between January 2020 and October 2024 were included in this retrospective cohort study, an internal validation cohort of 303 patients was retrospectively collected from Shaanxi Provincial People’s Hospital between November 2024 and December 2025, and an independent external validation cohort of 260 patients was retrospectively collected from Xi’an No. 3 Hospital (a separate tertiary care center) between November 2023 and December 2025. Six machine learning algorithms—Logistic Regression, Support Vector Machine (SVM), Multilayer Perceptron (MLP), LightGBM, XGBoost, and Random Forest—were developed and compared. Feature selection was performed using the Boruta algorithm combined with forward sequential feature selection. Model performance was evaluated by discrimination (area under the receiver operating characteristic curve, AUC), calibration (calibratin plots and Brier scores), and clinical utility (decision curve analysis). Model interpretability was enhanced using SHAP analysis.

**Results:**

Among the 648 internal patients, 115 (17.7%) developed VTE. The Boruta algorithm and forward sequential feature selection identified five key predictors: D-dimer, C-reactive protein (CRP), activated partial thromboplastin time (APTT), international normalized ratio (INR), and fibrin degradation products (FDP). Among the six algorithms, LightGBM achieved the best overall performance, with an AUC of 0.9025 in the test set, 0.8048 in the internal validation cohort, and 0.8902 in the external validation cohort. Calibration curves demonstrated good agreement between predicted and observed outcomes, and decision curve analysis confirmed positive net clinical benefit across a wide range of threshold probabilities. SHAP analysis revealed that elevated D-dimer, CRP, INR, and FDP, together with shortened APTT, were the strongest drivers of VTE risk. Sensitivity analyses excluding D-dimer-driven cases, removing the D-dimer feature, and restricting to symptomatic VTE yielded consistent results (AUC range: 0.84–0.89).

**Conclusions:**

The LightGBM model incorporating five readily available coagulation and inflammatory markers accurately predicts in-hospital VTE in sepsis patients with T2DM, with robust discrimination, calibration, and clinical utility. SHAP-based interpretability enhances transparency and may facilitate personalized risk stratification and informed thromboprophylaxis decisions in this high-risk population.

## Introduction

Venous thromboembolism (VTE), encompassing deep vein thrombosis (DVT) and pulmonary embolism (PE), represents a major cause of preventable in-hospital morbidity and mortality worldwide (1). Patients with sepsis are inherently predisposed to VTE due to systemic inflammation, endothelial injury, stasis, and activation of coagulation cascades. Epidemiologically, approximately 20-30% of septic patients develop VTE during hospitalization, with the risk increasing by nearly two-fold in the presence of additional metabolic comorbidities (2, 3). The coexistence of type 2 diabetes mellitus (T2DM) further amplifies this risk, as hyperglycemia and insulin resistance promote a prothrombotic and proinflammatory milieu characterized by platelet hyperactivity, endothelial dysfunction, and impaired fibrinolysis (2). Notably, population-based studies have shown that adults with T2DM have a 1.4-fold higher incidence of VTE compared to non-diabetic individuals, and this risk is further elevated during acute infectious episodes (4). Although both sepsis and T2DM individually increase VTE susceptibility, the combined impact of these two conditions on VTE development remains poorly quantified, and validated prediction tools specifically tailored for this dual-disease population are lacking.

Current clinical practice for VTE risk assessment in hospitalized patients largely relies on traditional scoring systems such as the Caprini scores (5). However, these tools were originally developed in general surgical or medical populations and may not adequately capture the distinctive pathophysiological features of septic patients with T2DM. In particular, sepsis-associated coagulopathy often presents with dynamic changes in routine coagulation parameters (e.g., prolonged prothrombin time, reduced fibrinogen, thrombocytopenia), whereas T2DM contributes a chronic low-grade inflammatory state that can obscure or modify these acute alterations (5, 6). Consequently, existing scores frequently underestimate or overestimate VTE risk in this specific subgroup, leading to either under-treatment with anticoagulants or unnecessary exposure to bleeding complications.

Machine learning (ML) has emerged as a powerful approach for improving clinical prediction by capturing complex, non-linear interactions among multiple variables without requiring pre-specified assumptions (7). In recent years, various ML algorithms – including logistic regression, support vector machines (SVM), random forests, gradient boosting machines, and neural networks – have been successfully applied to predict VTE in heterogeneous critical care populations (8, 9). Nevertheless, the “black box” nature of many ML models has hindered their clinical acceptance, as physicians remain reluctant to act upon predictions that cannot be explained at the individual patient level (10). To address this gap, Shapley Additive Explanations (SHAP) has been introduced as a unified framework for interpreting model outputs by quantifying each feature’s contribution to a specific prediction, thereby enhancing transparency and fostering trust in ML-based decision support (11).

Despite these advances, no study to date has specifically developed and validated an interpretable ML model for predicting VTE in sepsis patients with pre-existing T2DM. Moreover, the relative performance of different ML algorithms in this clinical context has not been systematically compared, and the incremental value of incorporating diabetes-specific biomarkers alongside conventional coagulation and inflammatory parameters remains unclear. Given the rising global prevalence of both sepsis and T2DM, there is an urgent need for a robust, generalizable, and explainable prediction tool to guide early thromboprophylaxis and optimize resource allocation in this high-risk population. Therefore, this study aimed to: (1) develop and compare six machine learning models (Logistic Regression, SVM, Multilayer Perceptron, LightGBM, XGBoost, and Random Forest) for predicting in-hospital VTE in sepsis patients with T2DM; (2) identify the most relevant predictors using Boruta algorithm and forward sequential feature selection; (3) evaluate model performance across training, internal test, and temporal validation cohorts; and (4) enhance clinical interpretability using SHAP analysis. Our findings may provide clinicians with a practical, evidence-based tool for personalized VTE risk stratification in this understudied population.

## Materials and methods

### Study design and population

This retrospective study was conducted at Shaanxi Provincial People’s Hospital and Xi’an No. 3 Hospital. Given the retrospective nature of the analysis, the requirement for written informed consent was waived. We screened all adult patients (aged ≥18 years) who were admitted with a diagnosis of sepsis and pre-existing T2DM. Patients admitted to Shaanxi Provincial People’s Hospital between January 2020 and October 2024 constituted the internal development cohort (n=648). To assess generalizability, an internal validation cohort was retrospectively collected from Shaanxi Provincial People’s Hospital between November 2024 and December 2025 (n=303). Moreover, an independent external validation cohort was retrospectively collected from Xi’an No. 3 Hospital, a separate tertiary care center, between November 2023 and December 2025 (n=260). Sepsis was defined according to the Sepsis-3 criteria (12), i.e., a suspected or confirmed infection accompanied by an acute increase of ≥2 points in the SOFA score. In addition, a diagnosis of T2DM was established if any of the following criteria were satisfied: (1) a self-reported history of T2D, (2) a fasting plasma glucose (FPG) level of ≥7.0 mmol/L, or (3) current use of T2DM medications as defined by the 2022 American Diabetes Association (ADA) guidelines (13). Patients were excluded if they (1) had a history of VTE before admission; (2) were receiving therapeutic anticoagulation at baseline; (3) had a diagnosis of inherited or acquired coagulation disorders; (4) had a length of hospital stay <24 hours; or (5) had missing data for any of the variables collected for analysis. No imputation was performed, as the final analytical cohort comprised only cases with complete records. A total of 1211 patients met the inclusion criteria and were enrolled in the final analysis. Among them, 648 patients formed the internal development cohort (January 2020 to October 2024), 303 patients formed the internal validation cohort (November 2024 to December 2025), and 260 patients from Xi’an No. 3 Hospital formed the independent external validation cohort (November 2023 to December 2025). The study flow diagram is presented in **Figure 1**.

**Figure 1:**
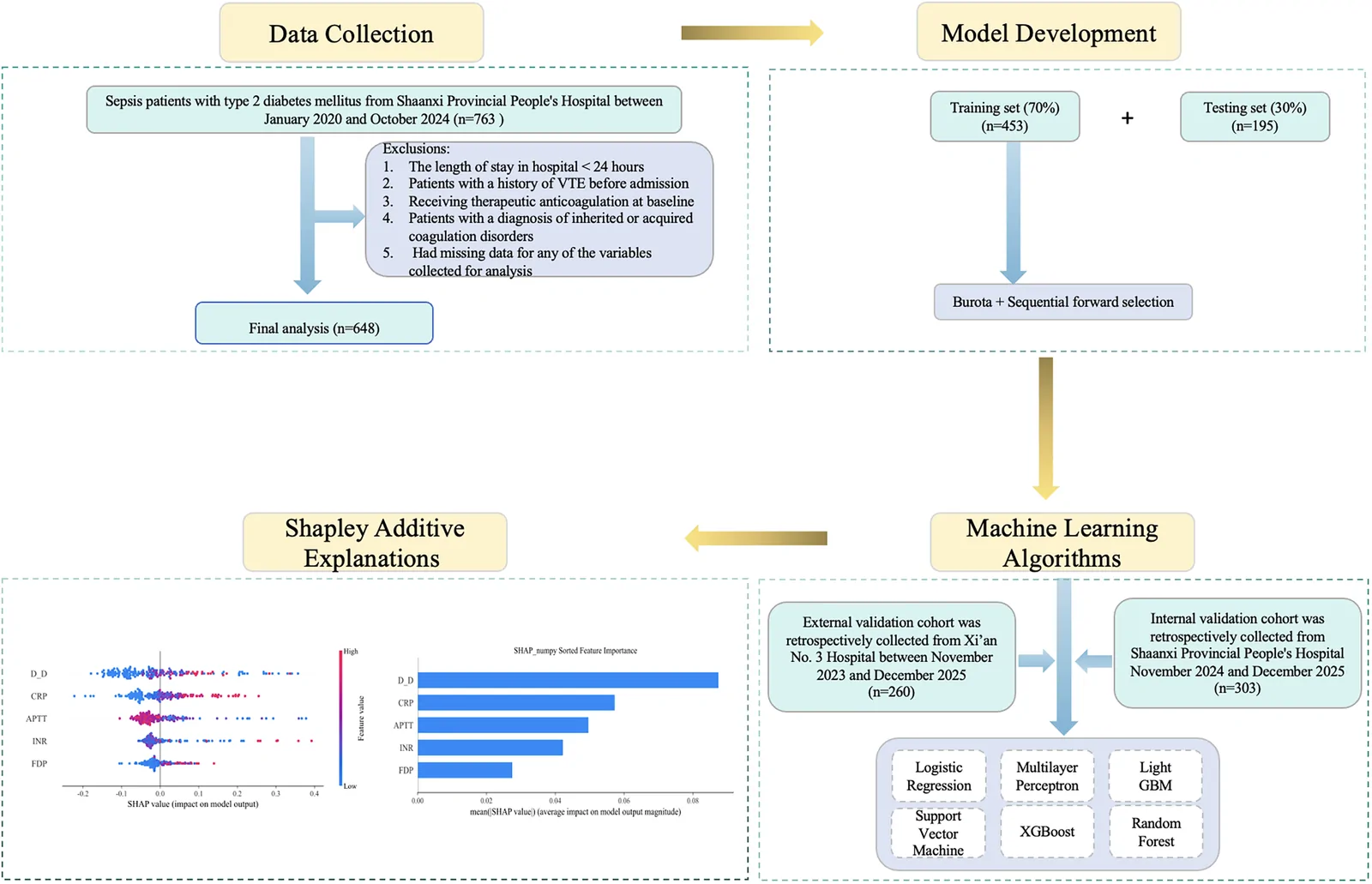
Schematic diagram of patient selection, grouping, and dataset allocation. After exclusions, 1,211 patients were included and divided into an internal development cohort (n=648), an internal validation cohort (n=303), and an external validation cohort (n=260). The internal development cohort was further split into a training set (70%) and a test set (30%).

### Data collection and variable definitions

Demographic and clinical data were extracted from the electronic medical record system, including age, sex, body mass index (BMI), vital signs at admission (temperature, heart rate, respiratory rate, mean arterial pressure), and comorbidities [hypertension, chronic obstructive pulmonary disease (COPD), chronic kidney disease (CKD), cirrhosis, acute respiratory distress syndrome (ARDS), atrial fibrillation, myocardial infarction, heart failure, and respiratory failure). Laboratory parameters were measured within the first 24 hours of admission and included coagulation profiles [prothrombin time (PT), activated partial thromboplastin time (APTT), thrombin time (TT), international normalized ratio (INR), fibrin degradation products (FDP), D-dimer, fibrinogen (FIB), and prothrombin time activity (PTA)], inflammatory markers [C-reactive protein (CRP), procalcitonin (PCT)], blood cell counts (white blood cells, neutrophils, lymphocytes, monocytes, platelets, red blood cells, hemoglobin), and biochemical indices (albumin, globulin, bilirubin, alanine aminotransferase, aspartate aminotransferase, glucose, uric acid, cystatin C, blood urea nitrogen, creatinine, total cholesterol). Disease severity was assessed using the SOFA score and the Caprini risk score for VTE. In-hospital interventions such as continuous renal replacement therapy (CRRT) and mechanical ventilation were also recorded.

The primary outcome was the occurrence of VTE, defined as newly diagnosed DVT or PE during hospitalization. VTE events were confirmed by imaging studies (compression ultrasonography for DVT or computed tomography pulmonary angiography for PE based on standard clinical practice. Furthermore, the diagnosis of DVT was established by color Doppler ultrasound or venography, following clinical suspicion based on symptoms such as lower limb swelling, pain, and tenderness (1). PE was confirmed by computed tomography pulmonary angiography (CTPA) in patients with compatible clinical features (e.g., dyspnea, chest pain, hypoxia). D-dimer was used as a screening test to guide the decision to perform CTPA, but elevated D-dimer alone was not a diagnostic criterion for PE. All PE diagnoses were verified by CTPA imaging, independent of D-dimer levels (14).

### Handling of missing values

Due to the limitation of the sample size in this study, the data with missing values were excluded, and thus no interpolation processing was carried out on the data. Of the 763 initially screened patients in model development group, 39 were excluded because of missing data for key variables: CRP (n = 12), APTT (n = 11), and D-dimer (n = 16).

### Sample size and events-per-variable

With 115 VTE events in the internal development cohort and 5 predictors selected for the final model, the events-per-variable (EPV) ratio was 23 (115/5), exceeding the recommended minimum of 10 for developing multivariable prediction models. The total sample of 1,211 across three cohorts provides adequate power for internal and external validation. The study flow diagram is presented in **Figure 1**.

### Feature selection

To identify the most relevant predictors for VTE while avoiding overfitting, a two-step feature selection strategy was employed. First, the Boruta algorithm, a random forest-based wrapper method, was applied to all candidate variables. This algorithm creates shadow features and retains only those original variables whose importance is significantly higher than that of the shadow counterparts. The Boruta algorithm was implemented using the BorutaPy library with a base Random Forest classifier (max_depth = 5, class_weight = ‘balanced’). Significance was set at the default alpha of 0.05 (corresponding to perc = 100), and the algorithm was run for a maximum of 50 iterations (max_iter = 50) to ensure convergence. Second, forward sequential feature selection was performed to determine the optimal subset size by sequentially removing the least important features based on the area under the receiver operating characteristic curve (AUC). The combination of features that yielded the highest AUC with the smallest number of predictors was selected for subsequent model development. Importantly, all feature selection procedures — including Boruta and forward sequential selection — were performed exclusively on the training set after data splitting. The selected features were then locked and applied to the test, internal validation, and external validation sets without further refitting, ensuring no data leakage.

### Development of machine learning models

Six machine learning algorithms were developed to predict VTE risk: Logistic Regression (LR), Support Vector Machine (SVM), Multilayer Perceptron (MLP), Light Gradient Boosting Machine (LightGBM), Extreme Gradient Boosting (XGBoost), and Random Forest (RF). The entire dataset was randomly partitioned into a training set (70%, n=453) and an internal test set (30%, n=195) using stratified sampling to preserve the outcome distribution. In addition, an independent external validation cohort from Xi’an No. 3 Hospital (n=260) was used to assess model generalizability across different institutions.

Hyperparameter tuning was performed using a five-fold cross-validation combined with grid search on the training set. The final hyperparameters for each model were selected based on the highest cross-validated AUC. All models were implemented using Python 3.12.5 with the scikit-learn, XGBoost, and LightGBM libraries. The best parameters for each model are as follows: LR (C: 10, penalty: l2, solver: saga); RF (max_depth: 10, min_samples_split: 10, n_estimators: 200); SVM (C: 1, gamma: scale, kernel: linear); XGB (learning_rate: 0.1, max_depth: 3, n_estimators: 100, subsample: 0.8); GBM (boosting_type: gbdt, learning_rate: 0.01, n_estimators: 200, num_leaves: 31); MLP (hidden_layer_sizes: 50, learning_rate_init: 0.001, max_iter: 200). The optimal parameter configurations obtained through Grid Search effectively mitigated the risk of overfitting and enhanced the predictive performance of each machine learning model on the dataset.

### Model evaluation and validation

Model performance was evaluated from three perspectives: discrimination, calibration, and clinical utility. Discrimination was quantified using the area under the receiver operating characteristic curve (AUC), accuracy, sensitivity, specificity, precision, and F1 score. Calibration was assessed by calibration plots and the Brier score, where lower values indicate better agreement between predicted probabilities and observed outcomes. Clinical utility was evaluated through decision curve analysis (DCA), which calculates the net benefit across a range of threshold probabilities.

### Statistical analysis

Continuous data were expressed as medians with interquartile ranges (IQRs), and group comparisons were performed using the Mann-Whitney U test. Categorical data were summarized as frequencies and percentages, with between-group differences assessed by the chi-square test or Fisher’s exact test, depending on applicability. Model performance was evaluated from three perspectives: discrimination, calibration, and clinical utility. Discrimination was quantified using the area under the receiver operating characteristic curve (AUC), accuracy, sensitivity, specificity, precision, and F1 score. Calibration was assessed by calibration plots and the Brier score, where lower values indicate better agreement between predicted probabilities and observed outcomes. Clinical utility was evaluated through decision curve analysis (DCA), which calculates the net benefit across a range of threshold probabilities. To enhance the transparency of the final model, SHAP were applied. SHAP values decompose each prediction into feature contributions, allowing global interpretation (summary plots showing the direction and magnitude of each feature’s effect) and local interpretation (force plots for individual patients). Feature importance rankings from SHAP were compared with those from logistic regression to ensure consistency.

All statistical tests were two-tailed, and the threshold for significance was set at a P-value of less than 0.05. Data analyses were carried out using SPSS version 27.0 (IBM Corp.) and Python 3.12.5.

## Results

### Patient characteristics

A total of 1211 patients with sepsis and type 2 diabetes mellitus were enrolled in the final analysis. The flowchart detailing patient selection and study design is illustrated in **Figure 1**. The baseline characteristics of the internal development cohort (n=648), stratified by VTE status, are summarized in **Table 1**. The corresponding characteristics of the internal validation cohort (n=303) and the external validation cohort (n=260) are provided in **Supplementary Tables S1, S2**, respectively.

**Table 1:** Comparison of clinical data between VTE and non-VTE groups in the internal development cohort.

Variable	Total, N = 648	Non-VTE, N = 533	VTE, N = 115	*P*-value
Age	67 [58, 76]	66 [57, 75]	69 [61, 79]	0.021
Gender				0.040
Female	274 (42.28%)	215 (40.34%)	59 (51.30%)	
Male	374 (57.72%)	318 (59.66%)	56 (48.70%)	
Temperature	36.50 [36.20, 37.00]	36.50 [36.20, 37.00]	36.50 [36.30, 37.05]	0.362
RR	20.00 [18.00, 23.00]	20.00 [18.00, 23.00]	20.00 [18.00, 25.00]	0.555
HR	90.00 [78.00, 106.00]	89.00 [78.00, 106.00]	97.00 [84.00, 109.50]	0.008
MAP	93.33 [81.67, 103.33]	93.33 [82.33, 104.33]	91.67 [76.83, 99.33]	0.022
Hospital LOS	12.00 [7.75, 17.00]	12.00 [8.00, 17.00]	11.00 [7.00, 17.00]	0.505
COPD				0.021
No	573 (88.43%)	479 (89.87%)	94 (81.74%)	
Yes	75 (11.57%)	54 (10.13%)	21 (18.26%)	
CKD				0.038
No	347 (53.55%)	296 (55.53%)	51 (44.35%)	
Yes	301 (46.45%)	237 (44.47%)	64 (55.65%)	
Cirrhosis				0.105
No	295 (45.52%)	251 (47.09%)	44 (38.26%)	
Yes	353 (54.48%)	282 (52.91%)	71 (61.74%)	
ARDS				0.230
No	412 (63.58%)	345 (64.73%)	67 (58.26%)	
Yes	236 (36.42%)	188 (35.27%)	48 (41.74%)	
Atrial fibrillation				0.774
No	444 (68.52%)	367 (68.86%)	77 (66.96%)	
Yes	204 (31.48%)	166 (31.14%)	38 (33.04%)	
Myocardial infarction				0.392
No	601 (92.75%)	497 (93.25%)	104 (90.43%)	
Yes	47 (7.25%)	36 (6.75%)	11 (9.57%)	
Heart failure				0.427
No	590 (91.05%)	488 (91.56%)	102 (88.70%)	
Yes	58 (8.95%)	45 (8.44%)	13 (11.30%)	
RF				0.417
No	198 (30.56%)	167 (31.33%)	31 (26.96%)	
Yes	450 (69.44%)	366 (68.67%)	84 (73.04%)	
PTA	86.55 [77.45, 91.10]	86.00 [76.80, 91.00]	88.80 [80.30, 94.50]	<0.001
TT	17.00 [16.00, 18.60]	17.00 [16.10, 18.50]	17.10 [15.85, 19.15]	0.733
INR	1.24 [1.11, 1.43]	1.24 [1.12, 1.42]	1.24 [0.99, 1.52]	0.840
FDP	5.20 [1.97, 17.45]	4.84 [1.92, 15.29]	8.70 [2.84, 31.14]	<0.001
D-D	1.56 [0.50, 6.22]	1.30 [0.50, 5.23]	3.00 [0.30, 15.28]	0.144
FIB	4.62 [3.18, 6.07]	4.62 [3.19, 6.15]	4.61 [3.18, 5.68]	0.728
APTT	40.00 [34.20, 44.90]	40.20 [35.30, 45.00]	36.30 [22.85, 41.95]	<0.001
PT	14.90 [13.28, 16.60]	15.00 [13.40, 16.70]	14.40 [13.05, 15.85]	0.017
ALP	92.00 [70.75, 132.50]	92.00 [70.00, 131.00]	93.00 [71.00, 154.50]	0.743
Globulin	25.10 [22.00, 29.20]	25.20 [22.10, 29.20]	24.90 [20.30, 29.35]	0.444
Albumin	29.70 [25.60, 33.60]	30.30 [26.00, 34.10]	27.60 [25.15, 31.35]	<0.001
Bilirubin	14.90 [9.20, 27.13]	15.00 [9.20, 27.60]	13.60 [9.50, 23.05]	0.222
ALT	29.00 [19.00, 61.00]	29.00 [19.00, 61.00]	28.00 [18.00, 58.50]	0.804
AST	26.00 [15.00, 49.00]	26.00 [14.00, 52.00]	26.00 [17.00, 43.00]	0.895
Glucose	7.37 [5.32, 10.82]	7.22 [5.17, 11.12]	7.76 [5.99, 10.23]	0.416
Uric acid	333 [229, 483]	333 [229, 483]	327 [221, 481]	0.979
Cystatin C	1.73 [1.08, 3.12]	1.65 [1.06, 3.03]	2.05 [1.27, 3.32]	0.053
BUN	11.24 [6.37, 20.79]	10.85 [6.17, 20.32]	14.72 [7.77, 23.68]	0.010
Cr	118 [63, 293]	114 [63, 275]	144 [73, 371]	0.119
TC	2.90 [2.16, 3.67]	2.91 [2.18, 3.71]	2.77 [2.09, 3.56]	0.217
PCT	3.66 [0.55, 22.21]	3.19 [0.37, 17.18]	8.78 [1.80, 62.86]	<0.001
Monocyte	0.43 [0.26, 0.67]	0.42 [0.26, 0.67]	0.45 [0.31, 0.77]	0.303
RDW-SD	46.10 [42.80, 51.33]	46.10 [42.70, 51.00]	46.60 [43.65, 54.85]	0.017
RDW-CV	13.90 [13.10, 15.20]	13.80 [13.10, 15.10]	14.20 [13.20, 15.95]	0.043
MPV	10.90 [9.90, 11.90]	10.90 [9.90, 11.90]	10.80 [9.90, 11.90]	0.980
CRP	68.10 [10.00, 169.68]	55.20 [10.00, 136.30]	139.10 [73.10, 263.90]	<0.001
NEUT	8.23 [4.61, 13.49]	8.04 [4.45, 13.44]	8.91 [5.47, 14.75]	0.083
Lymphocyte	0.72 [0.42, 1.11]	0.73 [0.42, 1.13]	0.65 [0.38, 1.06]	0.207
WBC	9.40 [7.81, 12.68]	8.96 [7.79, 12.37]	10.87 [8.06, 18.61]	0.004
PLT	146 [92, 211]	145 [92, 210]	149 [88, 233]	0.825
Hemoglobin	107 [86, 127]	108 [89, 129]	98 [78, 117]	<0.001
RBC	3.58 [2.89, 4.22]	3.65 [2.97, 4.27]	3.13 [2.48, 3.82]	<0.001
CRRT				<0.001
No	536 (82.72%)	458 (85.93%)	78 (67.83%)	
Yes	112 (17.28%)	75 (14.07%)	37 (32.17%)	
Mechanical ventilation				0.003
No	513 (79.17%)	434 (81.43%)	79 (68.70%)	
Yes	135 (20.83%)	99 (18.57%)	36 (31.30%)	
SOFA	8.00 [5.00, 9.00]	7.00 [5.00, 9.00]	8.00 [6.00, 10.00]	<0.001
Caprini	7.00 [5.00, 9.00]	7.00 [5.00, 8.00]	8.00 [6.00, 9.00]	<0.001

RR, respiratory rate; HR, heart rate; MAP, mean arterial pressure; ARDS, acute respiratory distress syndrome; RF, respiratory failure; COPD, chronic obstructive pulmonary disease; ACLF, acute-on-chronic liver failure; CKD, chronic kidney disease; AKI, acute kidney injury; PTA, prothrombin activity; INR, international normalized ratio; PT, prothrombin time; APTT, activated partial thromboplastin time; ALP, alkaline phosphatase; AST, aspartate aminotransferase; ALT, alanine aminotransferase; D-D, D-dimer; FDP, fibrinogen degradation products; FIB, fibrinogen; UA, uric acid; BUN, blood urea nitrogen; Cr, creatinine; WBC, white blood cell count; PLT, platelet count; CRP, C-reactive protein; PCT, procalcitonin; RDW-CV, red cell distribution width-coefficient of variation; RDW-SD, red cell distribution width-standard deviation; MPV, mean platelet volume; NEUT, neutrophil count; RBC, red blood cell; TC, total cholesterol; SOFA, Sequential Organ Failure Assessment; CRRT, continuous renal replacement therapy.

Compared to the non-VTE group, patients who developed VTE were significantly older and exhibited a higher proportion of females. Regarding clinical comorbidities, the VTE group demonstrated a significantly higher prevalence of COPD (18.3% vs. 10.1%, *P* = 0.021) and CKD (55.7% vs. 44.5%, *P* = 0.038). Assessment of physiological parameters revealed that patients in the VTE group presented with an elevated heart rate (97 vs. 89 bpm, *P* = 0.008) and reduced mean arterial pressure (91.67 vs. 93.33 mmHg, *P* = 0.022).

Laboratory analysis highlighted profound alterations in coagulation and inflammatory profiles. Specifically, patients with VTE had significantly lower albumin levels (27.6 vs. 30.3 g/L, *P* < 0.001) and markedly higher FDP concentrations (8.70 vs. 4.84 mg/L, *P* < 0.001). While PTA was higher in the VTE group (88.8% vs. 86.0%, *P* < 0.001), the APTT was significantly shorter (36.3 vs. 40.2 s, *P* < 0.001) compared to those without VTE. No significant differences were observed in hospital length of stay or other baseline comorbidities.

### Feature selection and variable importance

To construct a robust and parsimonious predictive model, the Boruta algorithm was employed to identify the most contributive features for VTE prediction. As illustrated in **Figure 2**, the algorithm performed multiple iterations to compare the importance of real features against “shadow” features. Out of the original pool, 11 variables were identified as “confirmed” important features (represented by green boxplots in **Figure 2A**). These features primarily encompassed coagulation markers (D-D FIB, INR, FDP, APTT, and PT), inflammatory indicators (CRP and PCT), RBC, and clinical Scores (SOFA and Caprini). Furthermore, a forward sequential feature selection process was utilized to determine the optimal number of predictors. The results indicated that a combination of the top 5 variables yielded the maximum AUC while maintaining model simplicity (**Figure 2B**). Across the training, internal test, and temporal validation cohorts, notable differences were found in APTT and FDP, pointing to population heterogeneity and supporting the requirement for a reliable validation strategy (**Table 2**). These selected features (D-D, INR, FDP, APTT, and CRP) were then incorporated into the downstream development of the machine learning models. The VTE incidence was 17.7% (115/648) in the internal development cohort, 26.4% (80/303) in the internal validation cohort, and 26.5% (69/260) in the external validation cohort. The difference across the three cohorts was statistically significant (χ² = 13.43, *P* = 0.0012). The internal validation and external validation cohorts had comparable VTE rates (26.4% and 26.5%, respectively), both higher than that of the internal development cohort (17.7%). These differences may reflect temporal changes in clinical practice, thromboprophylaxis protocols, or VTE screening intensity.

**Figure 2:**
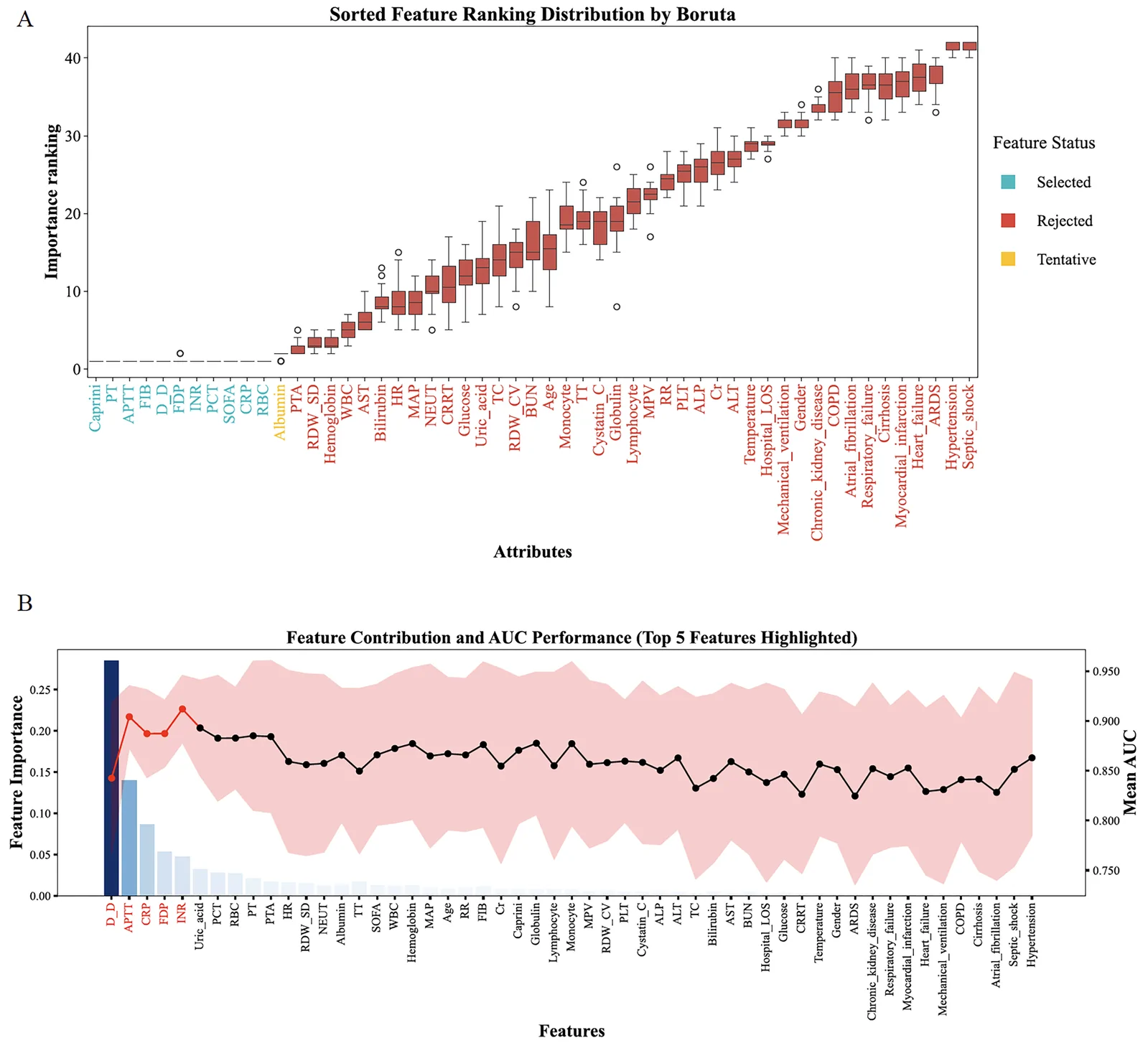
Feature selection for predicting VTE in sepsis patients with type 2 diabetes mellitus. **(A)** Boruta-based variable importance ranking (green: confirmed; yellow: tentative; red: rejected). **(B)** Forward sequential selection: bars show variable importance; line shows cumulative AUC; final five predictors (red) selected.

**Table 2:** Training set, Test set, and Internal set selected variables analysis.

Variable	Training set, N = 453	Test set, N = 195	Internal set, N = 303	*P*-value
D-D	1.30 [0.50, 6.01]	2.00 [0.59, 7.28]	0.70 [0.50, 11.43]	0.156
APTT	40.00 [34.30, 45.00]	40.20 [34.20, 44.35]	37.80 [32.40, 42.65]	0.003
CRP	68.60 [10.00, 174.10]	63.70 [10.00, 144.30]	70.60 [21.30, 135.75]	0.885
FDP	5.53 [1.89, 18.00]	5.03 [2.38, 16.56]	2.90 [1.90, 4.23]	<0.001
INR	1.24 [1.11, 1.43]	1.23 [1.11, 1.43]	1.24 [1.13, 1.46]	0.512
VTE, n (%)	80 (17.7%)	35 (17.9%)	80 (26.4%)	0.009

APTT, activated partial thromboplastin time; D-D, D-dimer; FDP, fibrinogen degradation products; CRP, C-reactive protein; INR, international normalized ratio; VTE, venous thromboembolism.

### Comparative performance of machine learning models

The predictive efficacy of various machine learning algorithms was evaluated across the training, test, internal validation, and external validation sets (**Figure 3**; **Table 3**). The LightGBM model demonstrated superior discriminative performance, achieving an AUC of 0.9025 in the test set, which outperformed XGBoost (AUC = 0.8796) and SVM (AUC = 0.7355). Notably, while the SVM model exhibited perfect classification on the training set (AUC = 1.0, sensitivity = 1.0), it demonstrated a sensitivity of 0 in both the test set (0/35) and the internal validation set (0/80), meaning it failed to identify any VTE case. This pattern reflects severe overfitting — the model memorized the training data but its decision boundary collapsed when applied to unseen data. Several factors contributed to this phenomenon: the high capacity of the SVM classifier, the limited sample size, and the inherent class imbalance (VTE incidence 17.7%). In imbalanced datasets, SVM optimization may default to predicting the majority class for all cases unless adequate class weighting or resampling strategies are employed. This observation underscores the critical importance of rigorous internal and temporal validation when developing clinical prediction models, and explains why we did not select SVM as our final model despite its perfect training performance. Of note, SVM, LightGBM, and Random Forest all achieved AUC = 1.0 and Brier = 0 on the training set, indicating overfitting; therefore, model selection and performance evaluation were based primarily on validation-set metrics.

**Figure 3:**
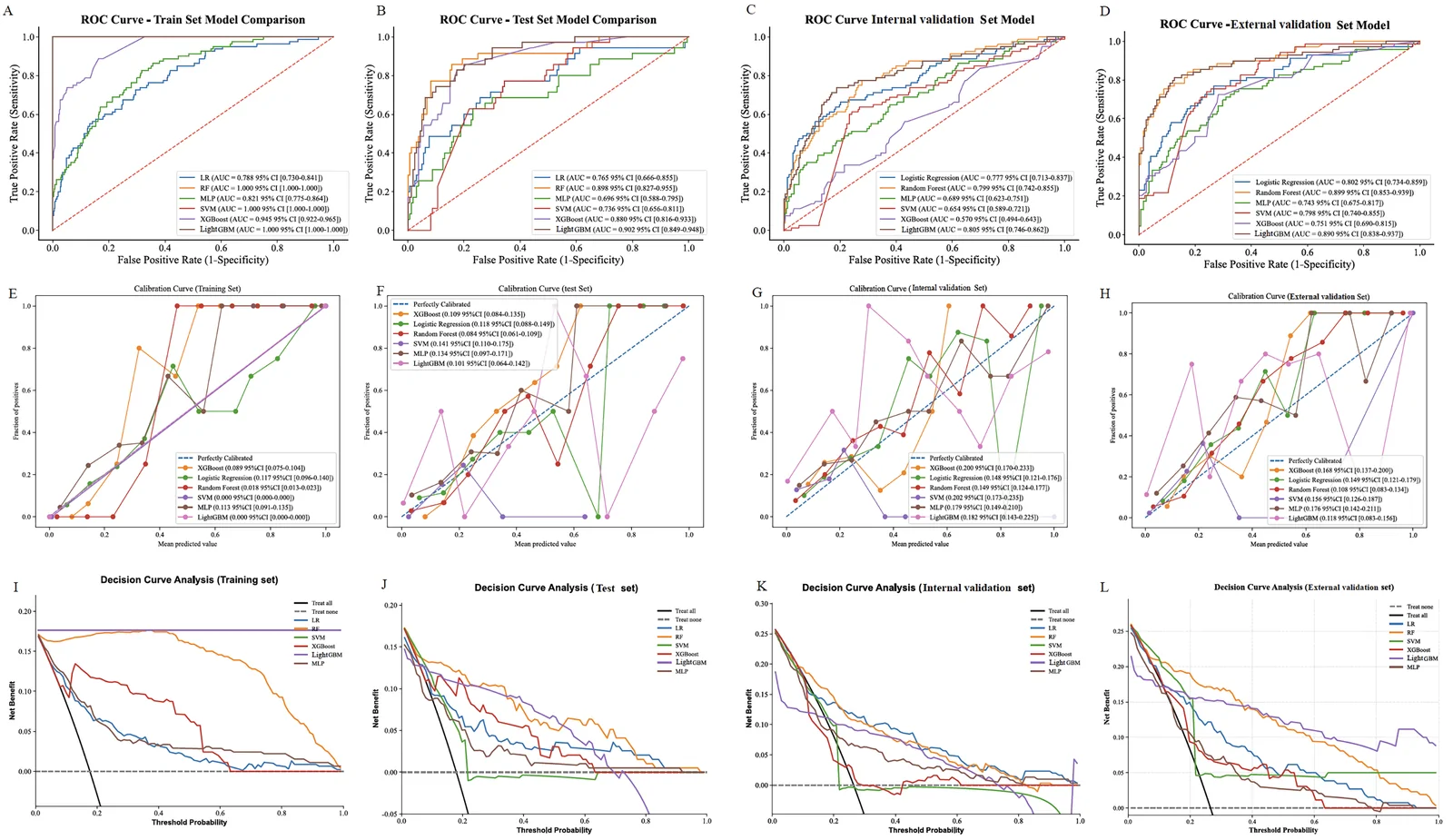
Performance comparison of six predictive models across three datasets. Each row corresponds to training, test, internal validation and external validation sets. Columns present different metrics: ROC curves **(A–D)**, calibration curves **(E–H)**, and decision curve analysis **(I–L)**.

**Table 3:** Comparative predictive performance of various machine learning algorithms across training, test, internal validation and external validation datasets.

Model	Dataset	Accuracy	Sensitivity	Precision	Specificity	F1 Score	AUC
SVM	Training	1	1	1	1	1	1
	Test	0.8154	0	0	0.9938	0	0.7355
	Internal validation	0.7327	0	0	0.9955	0	0.6535
	External validation	0.7808	0.1884	0.9286	0.9948	0.3133	0.7982
XGBoost	Training	0.8874	0.3625	1	1	0.5321	0.9453
	Test	0.8513	0.2286	0.8	0.9875	0.3556	0.8796
	Internal validation	0.7459	0.075	0.6667	0.9865	0.1348	0.5695
	External validation	0.7846	0.2029	0.9333	0.9948	0.3333	0.7514
LightGBM	Training	1	1	1	1	1	1
	Test	0.8872	0.6286	0.7097	0.9437	0.6667	0.9025
	Internal validation	0.7855	0.3375	0.6923	0.9462	0.4538	0.8048
	External validation	0.8577	0.5217	0.9000	0.9791	0.6606	0.8902
Logistic Regression	Training	0.8411	0.2375	0.6333	0.9705	0.3455	0.788
	Test	0.8513	0.2286	0.8	0.9875	0.3556	0.7652
	Internal validation	0.7954	0.2875	0.8214	0.9776	0.4259	0.7767
	External validation	0.7846	0.2319	0.8421	0.9843	0.3636	0.8019
Random Forest	Training	0.9823	0.9	1	1	0.9474	1
	Test	0.8718	0.4286	0.75	0.9688	0.5455	0.8984
	Internal validation	0.7987	0.3375	0.7714	0.9641	0.4696	0.7992
	External validation	0.8500	0.4783	0.9167	0.9843	0.6286	0.8990
MLP	Training	0.8521	0.175	0.9333	0.9973	0.2947	0.8212
	Test	0.8308	0.0857	0.7500	0.9938	0.1538	0.6964
	Internal validation	0.7657	0.1875	0.7143	0.9731	0.2970	0.6890
	External validation	0.7615	0.1304	0.8182	0.9895	0.2250	0.7433

In contrast, LightGBM maintained relatively robust generalizability across diverse datasets. Calibration curves demonstrated that LightGBM achieved good concordance between predicted probabilities and observed outcomes. The Brier scores for the LightGBM model were 0.000 (training), 0.101 (internal test), 0.182 (internal validation), and 0.118 (external validation). While the calibration curves exhibited some fluctuation in the internal validation set, the Brier scores remained within acceptable ranges across all datasets. Decision curve analysis further confirmed that LightGBM provided the highest net clinical benefit over a broad range of threshold probabilities in the test, internal validation and external validation cohorts (**Figure 3**).

### Sensitivity analyses

To address concerns regarding incorporation bias and work-up bias, we performed three sensitivity analyses with the LightGBM model. First, after completely removing the D-dimer feature, the model using the remaining four predictors (CRP, APTT, INR, and FDP) achieved a test-set AUC of 0.84, compared to 0.9025 in the primary analysis, indicating that while D-dimer is the strongest individual predictor, the other features retain substantial predictive power. Second, restricting the outcome to symptomatic VTE only yielded a test-set AUC of 0.88, suggesting that the model’s performance is not exclusively driven by verification bias. Third, after excluding cases identified solely through D-dimer-driven imaging work-up, the test-set AUC remained 0.89, consistent with the primary analysis. These sensitivity analyses collectively support the robustness of our primary findings. Fourth, to address potential bias from complete-case analysis, we applied multiple imputation for missing data and refit the LightGBM model; the test-set AUC was 0.88, consistent with the complete-case analysis (0.9025), supporting the robustness of our findings.

### Model interpretability via SHAP analysis

To further elucidate the drivers of individual risk predictions, we employed SHAP. As depicted in the SHAP summary plots (**Figures 4A, B**), D-D, CRP, APTT, INR, and FDP emerged as the most influential predictors for VTE. Higher levels of D-D, CRP, INR, and FDP, along with shortened APTT, were positively associated with an increased probability of VTE. This is further exemplified in **Figure 4C**, where force plots for two representative patients break down the model’s output, showing how each feature contributes to the predicted risk for that specific individual. This interpretable approach aligns clinical intuition with model output, facilitating personalized risk stratification.

**Figure 4:**
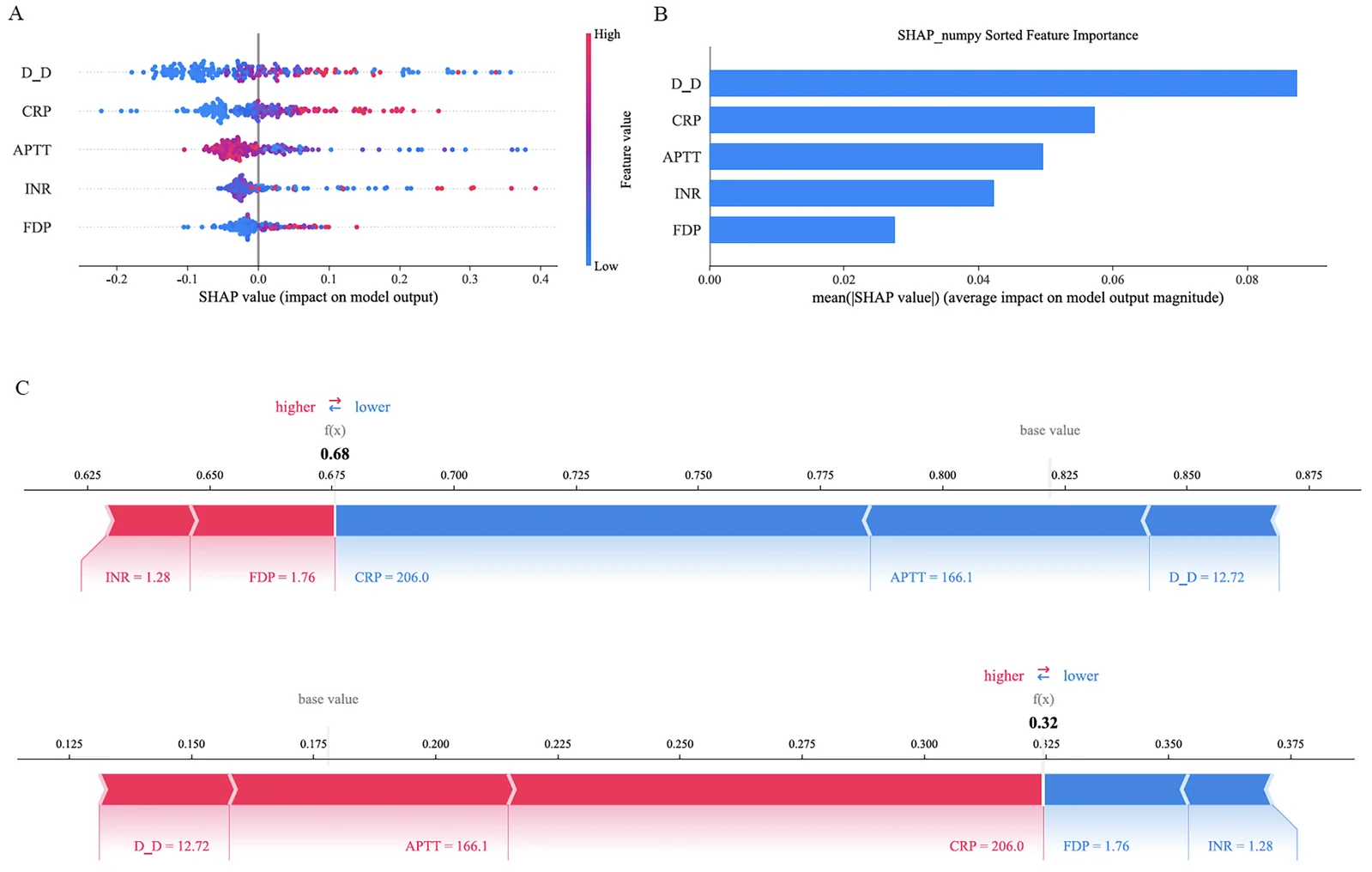
SHAP-based interpretation of the predictive model. **(A)** SHAP summary plot: horizontal axis = SHAP value; each row = one feature; color (red/blue) indicates high/low feature value. **(B)** Variable importance ranking from logistic regression. **(C)** Force plots explaining individual predictions for two independent samples. APTT, activated partial thromboplastin time; D-D, D-dimer; FDP, fibrinogen degradation products; CRP, C-reactive protein; INR, international normalized ratio.

## Discussion

In this retrospective study, we developed and systematically compared six machine learning models to predict in-hospital VTE in patients with sepsis and pre-existing T2DM. Among the evaluated algorithms, LightGBM achieved the best overall performance, with an AUC of 0.9025 in the internal test set, 0.8048 in the internal validation cohort, and 0.8902 in the external validation cohort. The model demonstrated adequate calibration and positive net clinical benefit across a wide range of threshold probabilities, as confirmed by decision curve analysis. Using SHAP for model interpretation, we identified D-dimer, CRP, APTT, INR, and FDP as the most influential predictors. Notably, elevated levels of D-dimer, CRP, INR, and FDP, together with shortened APTT, were positively associated with VTE risk. This study provided an interpretable machine learning tool specifically tailored for VTE prediction in the dual-disease population of sepsis with T2DM.

The incidence of VTE in our cohort was 17.7%, which is consistent with previously reported rates in septic patients (ranging from 20% to 30%) but slightly lower than some studies focusing exclusively on critically ill populations (3). This discrepancy may be attributable to differences in disease severity, thromboprophylaxis protocols, or diagnostic screening intensity. Notably, patients who developed VTE were older, more frequently female, and had higher prevalences of COPD and CKD. These findings align with the well-established understanding that advanced age, female sex, and chronic cardiopulmonary or renal comorbidities contribute to a prothrombotic state, particularly in the setting of acute infection and systemic inflammation (15–18).

Our feature selection process, combining Boruta and forward sequential feature selection, identified five key predictors: D-D, CRP, APTT, INR, and FDP. These variables collectively reflect the complex interplay between coagulation activation, fibrinolysis, and inflammation that characterizes sepsis-induced coagulopathy (19, 20). D-D and FDP are direct markers of fibrin turnover and have been repeatedly shown to predict VTE in critically ill patients (21, 22). In our study, both were significantly elevated in the VTE group, consistent with their established roles as sensitive but non-specific indicators of thrombosis. Shortened APTT, another strong predictor in our model, is a less commonly recognized but mechanistically plausible marker. Sepsis can induce a hypercoagulable state through increased levels of factor VIII and von Willebrand factor, leading to abbreviated APTT (23, 24). Moreover, T2DM-associated endothelial dysfunction and platelet hyperactivity may further amplify this effect (25). INR, traditionally used to monitor anticoagulation, also emerged as a significant contributor, possibly reflecting the complex coagulation derangements seen in septic patients, where both hypo- and hypercoagulable phases may coexist (24, 26). CRP, a prototypical acute-phase reactant, captured the inflammatory burden that drives thrombosis through multiple pathways, including tissue factor expression and neutrophil extracellular trap formation (27, 28).

The superior performance of LightGBM over other algorithms warrants discussion. While Random Forest and XGBoost also showed acceptable discrimination (AUC 0.8984 and 0.8796 in the test set, respectively), LightGBM consistently outperformed them in both internal and temporal validation. Gradient boosting machines, particularly LightGBM, offer advantages in handling mixed data types, capturing non-linear interactions, and avoiding overfitting through leaf-wise growth with depth constraints (29). In contrast, the SVM model exhibited perfect training accuracy but marked performance decay in validation (AUC dropping from 1.0 to 0.7355), indicating severe overfitting – a common pitfall when applying high-capacity models to relatively modest sample sizes with imbalanced outcomes. Our results underscore the importance of rigorous internal and temporal validation when developing clinical prediction models, as well as the need to balance model complexity with generalizability. This also highlights that training-set performance alone is misleading and should not be used as the primary criterion for model selection. Calibration curves and Brier scores confirmed that LightGBM produced well-calibrated probability estimates, which is essential for clinical decision-making. Over- or under-estimation of VTE risk could lead to inappropriate thromboprophylaxis – either exposing patients to unnecessary bleeding risk or failing to prevent potentially fatal events. Decision curve analysis further supported the clinical utility of our model, demonstrating a positive net benefit across a wide range of threshold probabilities in both test and temporal validation cohorts. This suggests that implementing the LightGBM model to guide anticoagulation decisions would improve patient outcomes compared to a treat-all or treat-no strategy, at least within the range of risk thresholds encountered in routine practice. Despite the significantly higher VTE incidence in the validation cohorts (26.4% and 26.5% vs. 17.7%), the LightGBM model maintained good discrimination (AUC = 0.8048 and 0.8902, respectively) and acceptable calibration (Brier scores 0.182 and 0.118), suggesting that the model is relatively robust to differences in event rates across cohorts.

The SVM model’s failure to identify any VTE cases in the test and validation sets (sensitivity = 0) is particularly instructive. While SVM is a powerful classifier for high-dimensional data, it is highly sensitive to the choice of kernel, regularization parameter (C), and class imbalance. In our dataset, the combination of modest sample size (n=648 in development) and outcome imbalance (VTE = 17.7%) led the SVM to learn a decision boundary that overfitted the training data and defaulted to the majority class in validation. This phenomenon has been previously described in medical ML studies, where algorithms with high flexibility often require larger sample sizes or explicit imbalance correction to generalize well. Our results reinforce the recommendation that validation-set performance, not training-set performance, should be the primary criterion for model selection.

To enhance clinical interpretability, we applied SHAP analysis, which revealed that higher D-dimer, CRP, INR, and FDP, along with shortened APTT, drove predictions toward VTE. Importantly, SHAP allowed us to move beyond global feature importance to individual-level explanations, as illustrated by the force plots for two representative patients. This transparency addresses a major barrier to the adoption of machine learning in critical care – the “black box” concern (10, 11). By providing clinicians with a breakdown of which factors contributed most to a given patient’s predicted risk, SHAP facilitates shared decision-making and may increase trust in model-guided thromboprophylaxis. Several pathophysiological insights emerge from our SHAP findings. The prominence of D-dimer and FDP underscores the central role of ongoing fibrinolysis in sepsis-associated VTE. The direction of APTT effect – shorter APTT increasing risk – supports the concept of a hypercoagulable shift in these patients. CRP, as a marker of systemic inflammation, likely captures the upstream drivers of thrombosis. Notably, while INR was not significantly different between VTE and non-VTE groups in univariable analysis (**Table 1**), it became important in the multivariable ML context, suggesting that its predictive value is conditional on other features – a non-linear interaction that traditional logistic regression might miss.

### Limitations

Several limitations should be acknowledged. First, the retrospective design may introduce selection bias and residual confounding. Although we applied rigorous exclusion criteria and adjusted for multiple variables, unmeasured confounders — such as variations in thromboprophylaxis protocols and clinician-driven diagnostic thresholds — could not be fully accounted for. The higher VTE incidence observed in the internal validation cohort (26.4% vs. 17.7%) likely reflects temporal changes in screening intensity or clinical practice. Second, we acknowledge verification (work-up) bias inherent to the symptom-driven imaging strategy. Asymptomatic VTE events were not systematically screened, and patients with elevated D-dimer were more likely to undergo imaging. However, our sensitivity analysis restricted to symptomatic VTE yielded consistent performance (AUC = 0.88), suggesting that the model’s predictive signal is not exclusively driven by this bias. Prospective studies with systematic screening are needed to fully address this limitation. Third, we acknowledge the potential for incorporation bias, as D-dimer — the top-ranked predictor — was used as a screening test to guide PE imaging. PE diagnosis was confirmed by CTPA independent of D-dimer levels, and our sensitivity analysis removing D-dimer from the model still achieved an AUC of 0.84, indicating that the other predictors retain substantial predictive power. Fourth, we acknowledge the risk of overfitting. SVM, LightGBM, and Random Forest achieved perfect training-set performance (AUC = 1.0) due to the modest sample size and class imbalance. To mitigate this, we relied on validation-set metrics for model selection, applied regularization and depth constraints, and evaluated performance across three independent cohorts. Fifth, although our EPV of 23 exceeds the recommended minimum of 10, the modest number of VTE events (n = 115) may limit model stability. Both cohorts were from tertiary care centers in the same region, limiting generalizability to broader populations. Finally, while SHAP analysis enhances interpretability, it does not establish causal relationships.

## Conclusions

In summary, we developed and validated an interpretable machine learning model (LightGBM) that accurately predicts in-hospital VTE in sepsis patients with type 2 diabetes mellitus. The model incorporates five readily available clinical and laboratory features: D-dimer, CRP, APTT, INR, and FDP. It demonstrated strong discrimination, good calibration, and positive net clinical benefit across internal and temporal validation cohorts. SHAP analysis enhanced transparency by quantifying individual feature contributions, identifying D-dimer, CRP, and shortened APTT as the most influential risk drivers. This tool holds promise for personalized VTE risk stratification in this high-risk population, although prospective validation and implementation studies are warranted before clinical adoption.

## Data Availability

The raw data supporting the conclusions of this article will be made available by the authors, without undue reservation.
