# Supplementary material for: Interpretable machine learning models to predict venous thromboembolism in sepsis patients with type 2 diabetes mellitus
Source: Front Endocrinol (Lausanne). 2026 Sep 30;17:1958305. doi: 10.3389/fendo.2026.1958305 (PMC13661359; doi:10.3389/fendo.2026.1958305)
Supplement: Supplementary file 1 [file Table1.docx]

**Supplementary Table S1.** Comparison of clinical data between VTE and non-VTE groups in the internal validation cohort.

| **Variable** | **Total, N = 303** | **Non-VTE, N = 223** | **VTE, N = 80** | ***P*-value** |
| --- | --- | --- | --- | --- |
| Age | 66.00 [55.00, 74.00] | 65.00 [53.00, 73.00] | 69.00 [60.50, 76.00] | 0.014 |
| Gender |  |  |  | 0.026 |
| Female | 163 (53.80%) | 129 (57.85%) | 34 (42.50%) |  |
| Male | 140 (46.20%) | 94 (42.15%) | 46 (57.50%) |  |
| Temperature | 36.50 [36.20, 36.80] | 36.50 [36.20, 36.80] | 36.50 [36.20, 36.85] | 0.548 |
| RR | 20.00 [19.00, 22.00] | 20.00 [19.00, 22.00] | 20.00 [18.50, 23.00] | 0.454 |
| HR | 88.00 [75.00, 103.00] | 87.00 [75.00, 101.00] | 94.00 [78.50, 105.00] | 0.079 |
| MAP | 93.33 [83.00, 105.00] | 92.67 [83.33, 104.67] | 98.50 [82.17, 106.00] | 0.413 |
| Hospital LOS | 12.00 [8.00, 17.00] | 11.00 [8.00, 16.00] | 14.00 [9.00, 20.00] | 0.103 |
| COPD |  |  |  | 0.956 |
| No | 260 (85.81%) | 192 (86.10%) | 68 (85.00%) |  |
| Yes | 43 (14.19%) | 31 (13.90%) | 12 (15.00%) |  |
| CKD |  |  |  | 0.160 |
| No | 171 (56.44%) | 120 (53.81%) | 51 (63.75%) |  |
| Yes | 132 (43.56%) | 103 (46.19%) | 29 (36.25%) |  |
| Cirrhosis |  |  |  | 0.222 |
| No | 147 (48.51%) | 103 (46.19%) | 44 (55.00%) |  |
| Yes | 156 (51.49%) | 120 (53.81%) | 36 (45.00%) |  |
| ARDS |  |  |  | >0.999 |
| No | 203 (67.00%) | 149 (66.82%) | 54 (67.50%) |  |
| Yes | 100 (33.00%) | 74 (33.18%) | 26 (32.50%) |  |
| Atrial fibrillation |  |  |  | 0.987 |
| No | 218 (71.95%) | 161 (72.20%) | 57 (71.25%) |  |
| Yes | 85 (28.05%) | 62 (27.80%) | 23 (28.75%) |  |
| Myocardial infarction |  |  |  | 0.962 |
| No | 275 (90.76%) | 203 (91.03%) | 72 (90.00%) |  |
| Yes | 28 (9.24%) | 20 (8.97%) | 8 (10.00%) |  |
| Heart failure |  |  |  | 0.689 |
| No | 267 (88.12%) | 198 (88.79%) | 69 (86.25%) |  |
| Yes | 36 (11.88%) | 25 (11.21%) | 11 (13.75%) |  |
| RF |  |  |  | 0.377 |
| YES | 207 (68.32%) | 156 (69.96%) | 51 (63.75%) |  |
| No | 96 (31.68%) | 67 (30.04%) | 29 (36.25%) |  |
| PTA | 80.32 (22.27) | 81.16 (21.49) | 77.97 (24.31) | 0.302 |
| TT | 16.90 [15.70, 18.30] | 16.90 [15.60, 18.50] | 16.65 [15.75, 18.05] | 0.514 |
| INR | 1.24 [1.13, 1.46] | 1.25 [1.15, 1.41] | 1.23 [0.98, 2.47] | 0.919 |
| FDP | 2.90 [1.90, 4.23] | 2.71 [1.98, 3.56] | 31.86 [1.89, 44.04] | <0.001 |
| D-D | 0.70 [0.50, 11.43] | 0.70 [0.47, 10.00] | 7.64 [0.54, 13.90] | 0.015 |
| FIB | 4.78 [3.39, 5.98] | 4.78 [3.41, 6.00] | 4.77 [3.32, 5.96] | 0.707 |
| APTT | 37.80 [32.40, 42.70] | 38.60 [33.40, 43.30] | 35.40 [24.00, 40.75] | 0.004 |
| PT | 14.50 [12.60, 15.90] | 15.00 [13.90, 16.10] | 1.45 [1.19, 12.85] | <0.001 |
| ALP | 90.00 [69.00, 135.00] | 91.00 [70.00, 139.00] | 87.00 [64.50, 126.50] | 0.364 |
| Globulin | 26.90 [23.30, 30.20] | 26.90 [23.40, 30.30] | 27.70 [23.00, 30.10] | 0.992 |
| Albumin | 31.10 [26.60, 35.70] | 31.10 [26.70, 36.30] | 31.10 [26.30, 34.00] | 0.427 |
| Bilirubin | 13.70 [8.60, 22.90] | 13.70 [8.40, 22.50] | 13.45 [9.25, 26.85] | 0.674 |
| ALT | 26.00 [18.00, 42.00] | 26.00 [18.00, 42.00] | 25.00 [17.00, 41.00] | 0.468 |
| AST | 22.00 [15.00, 38.00] | 23.00 [15.00, 38.00] | 21.00 [15.00, 38.50] | 0.626 |
| Glucose | 6.82 [5.41, 10.52] | 6.71 [5.21, 10.56] | 7.47 [5.75, 10.42] | 0.331 |
| Uric acid | 322.00 [233.00, 427.00] | 317.00 [242.00, 429.00] | 336.50 [218.50, 425.00] | 0.862 |
| Cystatin C | 1.50 [1.08, 3.08] | 1.47 [1.08, 3.12] | 1.69 [1.06, 3.07] | 0.647 |
| BUN | 9.07 [5.71, 18.26] | 8.95 [5.54, 17.26] | 9.67 [6.66, 19.38] | 0.281 |
| Cr | 111.00 [62.00, 265.00] | 105.00 [62.00, 266.00] | 122.50 [57.50, 239.50] | 0.947 |
| TC | 3.24 [2.26, 4.04] | 3.23 [2.26, 4.03] | 3.29 [2.23, 4.05] | 0.588 |
| PCT | 1.53 [0.29, 8.11] | 1.71 [0.30, 11.10] | 1.11 [0.29, 4.56] | 0.311 |
| Monocyte | 0.44 [0.28, 0.67] | 0.45 [0.28, 0.67] | 0.39 [0.24, 0.73] | 0.541 |
| RDW_SD | 47.40 [44.30, 52.10] | 47.10 [43.90, 52.10] | 48.55 [44.45, 51.65] | 0.159 |
| RDW_CV | 14.00 [13.20, 15.40] | 14.00 [13.10, 15.50] | 14.30 [13.50, 15.35] | 0.126 |
| MPV | 10.40 [9.60, 11.60] | 10.40 [9.50, 11.60] | 10.40 [9.70, 11.40] | 0.998 |
| CRP | 70.60 [20.80, 135.90] | 64.40 [20.30, 123.00] | 114.30 [28.20, 169.95] | 0.014 |
| NEUT | 7.26 [4.47, 12.46] | 7.19 [4.47, 11.91] | 7.63 [4.53, 14.18] | 0.399 |
| Lymphocyte | 0.71 [0.42, 1.11] | 0.73 [0.43, 1.11] | 0.60 [0.39, 1.09] | 0.161 |
| WBC | 9.03 [5.88, 14.06] | 7.98 [5.60, 12.81] | 10.41 [7.06, 15.10] | 0.012 |
| PLT | 166.00 [106.00, 254.00] | 173.00 [108.00, 265.00] | 145.00 [93.50, 226.00] | 0.098 |
| Hemoglobin | 101.00 [84.00, 124.00] | 103.00 [84.00, 127.00] | 97.50 [81.50, 117.50] | 0.211 |
| RBC | 3.40 [2.89, 4.09] | 3.48 [2.95, 4.15] | 3.24 [2.70, 3.84] | 0.065 |
| CRRT |  |  |  | 0.300 |
| No | 209 (68.98%) | 158 (70.85%) | 51 (63.75%) |  |
| Yes | 94 (31.02%) | 65 (29.15%) | 29 (36.25%) |  |
| Mechanical ventilation |  |  |  | 0.117 |
| YES | 98 (32.34%) | 66 (29.60%) | 32 (40.00%) |  |
| No | 205 (67.66%) | 157 (70.40%) | 48 (60.00%) |  |
| Caprini | 7.00 [5.00, 9.00] | 7.00 [5.00, 8.00] | 9.00 [7.00, 10.00] | <0.001 |
| SOFA | 8.00 [5.00, 9.00] | 7.00 [5.00, 8.00] | 9.00 [7.00, 10.50] | <0.001 |

Respiratory rate (RR), heart rate (HR), mean arterial pressure (MAP), acute respiratory distress syndrome (ARDS), respiratory failure (RF), chronic obstructive pulmonary disease (COPD), Acute-on-chronic liver failure (ACFL), chronic kidney disease (CKD), acute kidney injury (AKI), prothrombin activity (PTA), international normalized ratio (INR), prothrombin time (PT), activated partial thromboplastin time (APTT), alkaline phosphatase (ALP), aspartate aminotransferase (AST), alanine aminotransferase (ALT), D-dimer (D-D), fibrinogen degradation products (FDP), fibrinogen (FIB), uric acid (UA), blood urea nitrogen (BUN), creatinine (Cr), white blood cell count (WBC), platelet count (PLT), C-reactive protein (CRP), procalcitonin (PCT), red cell distribution width-coefficient of variation (RDW-CV), red cell distribution width-standard deviation (RDW-SD), mean platelet volume (MPV), neutrophil count (NEUT), red blood cell (RBC), lymphocyte count, total cholesterol (TC), Sequential Organ Failure Assessment (SOFA), continuous renal replacement therapy (CRRT).

**Supplementary Table S2.** Comparison of clinical data between VTE and non-VTE groups in the external validation cohort.

| **Variable** | **Total, N = 260** | **Non-VTE, N = 191** | **VTE, N = 69** | ***P*-value** |
| --- | --- | --- | --- | --- |
| Age | 68.00 [56.50, 76.00] | 67.00 [54.00, 76.00] | 69.00 [63.00, 79.00] | 0.030 |
| Gender |  |  |  | 0.022 |
| Male | 149 (57.31%) | 118 (61.78%) | 31 (44.93%) |  |
| Female | 111 (42.69%) | 73 (38.22%) | 38 (55.07%) |  |
| Temperature | 36.50 [36.20, 37.00] | 36.50 [36.20, 37.00] | 36.50 [36.30, 37.00] | 0.446 |
| RR | 20.00 [19.00, 23.00] | 20.00 [19.00, 23.00] | 21.00 [18.00, 24.00] | 0.380 |
| HR | 90.00 [79.00, 105.00] | 88.00 [78.00, 103.00] | 98.00 [81.00, 105.00] | 0.145 |
| MAP | 91.87 (16.66) | 92.78 (16.83) | 89.35 (16.01) | 0.135 |
| Hospital LOS | 12.00 [8.00, 16.00] | 11.00 [8.00, 16.00] | 13.00 [8.00, 19.00] | 0.317 |
| COPD |  |  |  | 0.618 |
| No | 225 (86.54%) | 167 (87.43%) | 58 (84.06%) |  |
| Yes | 35 (13.46%) | 24 (12.57%) | 11 (15.94%) |  |
| CKD |  |  |  | 0.840 |
| No | 144 (55.38%) | 107 (56.02%) | 37 (53.62%) |  |
| Yes | 116 (44.62%) | 84 (43.98%) | 32 (46.38%) |  |
| Cirrhosis |  |  |  | >0.999 |
| No | 124 (47.69%) | 91 (47.64%) | 33 (47.83%) |  |
| Yes | 136 (52.31%) | 100 (52.36%) | 36 (52.17%) |  |
| ARDS |  |  |  | 0.578 |
| No | 171 (65.77%) | 128 (67.02%) | 43 (62.32%) |  |
| Yes | 89 (34.23%) | 63 (32.98%) | 26 (37.68%) |  |
| Atrial fibrillation |  |  |  | 0.761 |
| No | 179 (68.85%) | 133 (69.63%) | 46 (66.67%) |  |
| Yes | 81 (31.15%) | 58 (30.37%) | 23 (33.33%) |  |
| Myocardial infarction |  |  |  | >0.999 |
| No | 234 (90.00%) | 172 (90.05%) | 62 (89.86%) |  |
| Yes | 26 (10.00%) | 19 (9.95%) | 7 (10.14%) |  |
| Heart failure |  |  |  | 0.906 |
| No | 229 (88.08%) | 169 (88.48%) | 60 (86.96%) |  |
| Yes | 31 (11.92%) | 22 (11.52%) | 9 (13.04%) |  |
| PTA | 86.00 [75.50, 92.40] | 86.00 [76.10, 92.00] | 86.10 [73.00, 95.00] | 0.836 |
| TT | 17.05 [16.00, 18.60] | 17.00 [16.00, 18.80] | 17.10 [15.80, 18.30] | 0.517 |
| INR | 1.25 [1.13, 1.43] | 1.26 [1.15, 1.41] | 1.23 [0.98, 2.38] | 0.883 |
| FDP | 3.51 [1.96, 19.54] | 3.18 [1.97, 7.07] | 22.50 [1.92, 39.31] | <0.001 |
| D_D | 1.71 [0.50, 9.78] | 0.70 [0.50, 8.08] | 4.36 [0.50, 12.65] | 0.083 |
| FIB | 4.85 (2.10) | 4.82 (2.21) | 4.93 (1.79) | 0.679 |
| APTT | 38.40 [32.70, 43.25] | 38.80 [35.10, 44.30] | 35.60 [26.40, 41.20] | 0.002 |
| PT | 14.60 [13.00, 15.90] | 15.00 [13.80, 16.20] | 13.00 [1.27, 14.70] | <0.001 |
| ALP | 90.00 [72.00, 148.00] | 90.00 [71.00, 151.00] | 93.00 [75.00, 135.00] | 0.672 |
| Globulin | 26.15 [22.90, 29.85] | 26.10 [23.00, 30.30] | 26.20 [22.00, 28.90] | 0.397 |
| Bilirubin | 15.45 [9.05, 26.85] | 15.60 [9.10, 26.20] | 14.30 [9.00, 28.90] | 0.779 |
| Albumin | 29.85 [25.50, 34.35] | 30.20 [25.60, 35.00] | 28.00 [25.20, 32.90] | 0.153 |
| ALT | 28.00 [19.00, 52.50] | 28.00 [19.00, 53.00] | 28.00 [20.00, 51.00] | 0.786 |
| AST | 25.00 [17.00, 44.50] | 25.00 [17.00, 44.00] | 27.00 [19.00, 45.00] | 0.528 |
| Glucose | 7.79 [5.38, 10.65] | 7.46 [5.13, 10.57] | 8.35 [6.43, 10.78] | 0.058 |
| Uric acid | 323.00 [233.50, 439.00] | 319.00 [236.00, 437.00] | 349.00 [225.00, 442.00] | 0.988 |
| Cystatin C | 1.70 [1.11, 3.08] | 1.53 [1.06, 2.71] | 2.18 [1.27, 3.34] | 0.029 |
| BUN | 10.64 [6.45, 19.54] | 9.75 [5.98, 17.89] | 13.23 [7.39, 21.30] | 0.022 |
| Cr | 117.50 [65.00, 251.50] | 108.00 [64.00, 224.00] | 166.00 [72.00, 293.00] | 0.083 |
| TC | 2.99 [2.19, 3.80] | 2.98 [2.20, 3.80] | 2.99 [2.16, 3.74] | 0.744 |
| PCT | 2.51 [0.53, 14.83] | 2.32 [0.44, 11.09] | 2.80 [0.62, 30.41] | 0.261 |
| Monocyte | 0.42 [0.26, 0.69] | 0.43 [0.26, 0.66] | 0.39 [0.22, 0.78] | 0.661 |
| RDW_SD | 47.00 [43.80, 52.15] | 46.60 [43.40, 52.10] | 48.80 [44.90, 53.40] | 0.027 |
| RDW_CV | 14.00 [13.20, 15.20] | 14.00 [13.10, 15.20] | 14.10 [13.40, 15.20] | 0.240 |
| MPV | 10.70 [9.70, 11.80] | 10.70 [9.70, 11.70] | 10.70 [9.70, 12.10] | 0.766 |
| NEUT | 7.99 [4.90, 13.76] | 7.78 [4.77, 13.43] | 9.03 [5.78, 15.02] | 0.289 |
| Lymphocyte | 0.69 [0.38, 1.10] | 0.72 [0.41, 1.12] | 0.61 [0.30, 0.95] | 0.031 |
| WBC | 8.84 [6.84, 12.74] | 8.22 [6.58, 12.13] | 9.90 [7.82, 18.18] | 0.029 |
| PLT | 149.50 [88.50, 227.00] | 155.00 [89.00, 233.00] | 145.00 [86.00, 218.00] | 0.431 |
| Hemoglobin | 103.00 [86.50, 128.50] | 107.00 [89.00, 130.00] | 98.00 [84.00, 120.00] | 0.101 |
| RBC | 3.42 [2.86, 4.19] | 3.64 [2.95, 4.29] | 3.17 [2.67, 3.99] | 0.014 |
| CRRT |  |  |  | <0.001 |
| No | 190 (73.08%) | 151 (79.06%) | 39 (56.52%) |  |
| Yes | 70 (26.92%) | 40 (20.94%) | 30 (43.48%) |  |
| Mechanical ventilation |  |  |  | 0.011 |
| YES | 73 (28.08%) | 45 (23.56%) | 28 (40.58%) |  |
| No | 187 (71.92%) | 146 (76.44%) | 41 (59.42%) |  |
| SOFA | 8.00 [5.00, 9.00] | 7.00 [5.00, 8.00] | 9.00 [7.00, 12.00] | <0.001 |
| Caprini | 7.00 [5.00, 9.00] | 7.00 [5.00, 8.00] | 8.00 [7.00, 9.00] | <0.001 |

Respiratory rate (RR), heart rate (HR), mean arterial pressure (MAP), acute respiratory distress syndrome (ARDS), respiratory failure (RF), chronic obstructive pulmonary disease (COPD), Acute-on-chronic liver failure (ACFL), chronic kidney disease (CKD), acute kidney injury (AKI), prothrombin activity (PTA), international normalized ratio (INR), prothrombin time (PT), activated partial thromboplastin time (APTT), alkaline phosphatase (ALP), aspartate aminotransferase (AST), alanine aminotransferase (ALT), D-dimer (D-D), fibrinogen degradation products (FDP), fibrinogen (FIB), uric acid (UA), blood urea nitrogen (BUN), creatinine (Cr), white blood cell count (WBC), platelet count (PLT), C-reactive protein (CRP), procalcitonin (PCT), red cell distribution width-coefficient of variation (RDW-CV), red cell distribution width-standard deviation (RDW-SD), mean platelet volume (MPV), neutrophil count (NEUT), red blood cell (RBC), lymphocyte count, total cholesterol (TC), Sequential Organ Failure Assessment (SOFA), continuous renal replacement therapy (CRRT).
